# Phenotypic and Transcriptomic Analyses of Seven Clinical Stenotrophomonas maltophilia Isolates Identify a Small Set of Shared and Commonly Regulated Genes Involved in the Biofilm Lifestyle

**DOI:** 10.1128/AEM.02038-20

**Published:** 2020-11-24

**Authors:** Ifey Alio, Mirja Gudzuhn, Pablo Pérez García, Dominik Danso, Marie Charlotte Schoelmerich, Uwe Mamat, Ulrich E. Schaible, Jörg Steinmann, Daniel Yero, Isidre Gibert, Thomas A. Kohl, Stefan Niemann, Matthias I. Gröschel, Johanna Haerdter, Thomas Hackl, Christel Vollstedt, Mechthild Bömeke, Richard Egelkamp, Rolf Daniel, Anja Poehlein, Wolfgang R. Streit

**Affiliations:** a Department of Microbiology and Biotechnology, Universität Hamburg, Hamburg, Germany; b Molecular and Experimental Mycobacteriology, Priority Area Infections, Research Center Borstel, Borstel, Germany; c Institut für Klinikhygiene, Medizinische Mikrobiologie und Klinische Infektiologie, Universitätsinstitut der Paracelsus Medizinischen Privatuniversität Klinikum Nürnberg, Nürnberg, Germany; d Department of Genetics and Microbiology and Institute of Biotechnology and Biomedicine, Universitat Autònoma de Barcelona, Barcelona, Spain; e Molecular and Experimental Mycobacteriology, Research Center Borstel, Borstel, Germany; f Department of Biomedical Informatics, Harvard Medical School, Boston, Massachusetts, USA; g Institute of Organic Chemistry, Universität Hamburg, Hamburg, Germany; h Institute of Microbiology and Genetics, Department of Genomic and Applied Microbiology, Georg-August University of Göttingen, Göttingen, Germany; North Carolina State University

**Keywords:** *Stenotrophomonas*, transcriptome, biofilms

## Abstract

Stenotrophomonas maltophilia is one of the most frequently isolated multidrug-resistant nosocomial opportunistic pathogens. It contributes to disease progression in cystic fibrosis (CF) patients and is frequently isolated from wounds, infected tissues, and catheter surfaces. On these diverse surfaces S. maltophilia lives in single-species or multispecies biofilms. Since very little is known about common processes in biofilms of different S. maltophilia isolates, we analyzed the biofilm profiles of 300 clinical and environmental isolates from Europe of the recently identified main lineages Sgn3, Sgn4, and Sm2 to Sm18. The analysis of the biofilm architecture of 40 clinical isolates revealed the presence of multicellular structures and high phenotypic variability at a strain-specific level. Further, transcriptome analyses of biofilm cells of seven clinical isolates identified a set of 106 shared strongly expressed genes and 33 strain-specifically expressed genes. Surprisingly, the transcriptome profiles of biofilm versus planktonic cells revealed that just 9.43% ± 1.36% of all genes were differentially regulated. This implies that just a small set of shared and commonly regulated genes is involved in the biofilm lifestyle. Strikingly, iron uptake appears to be a key factor involved in this metabolic shift. Further, metabolic analyses implied that S. maltophilia employs a mostly fermentative growth mode under biofilm conditions. The transcriptome data of this study together with the phenotypic and metabolic analyses represent so far the largest data set on S. maltophilia biofilm versus planktonic cells. This study will lay the foundation for the identification of strategies for fighting S. maltophilia biofilms in clinical and industrial settings.

**IMPORTANCE** Microorganisms living in a biofilm are much more tolerant to antibiotics and antimicrobial substances than planktonic cells are. Thus, the treatment of infections caused by microorganisms living in biofilms is extremely difficult. Nosocomial infections (among others) caused by S. maltophilia, particularly lung infection among CF patients, have increased in prevalence in recent years. The intrinsic multidrug resistance of S. maltophilia and the increased tolerance to antimicrobial agents of its biofilm cells make the treatment of S. maltophilia infection difficult. The significance of our research is based on understanding the common mechanisms involved in biofilm formation of different S. maltophilia isolates, understanding the diversity of biofilm architectures among strains of this species, and identifying the differently regulated processes in biofilm versus planktonic cells. These results will lay the foundation for the treatment of S. maltophilia biofilms.

## Abstract (precis)

Microorganisms living in a biofilm are much more tolerant to antibiotics and antimicrobial substances than planktonic cells are. Thus, the treatment of infections caused by microorganisms living in biofilms is extremely difficult. Nosocomial infections (among others) caused by S. maltophilia, particularly lung infection among CF patients, have increased in prevalence in recent years. The intrinsic multidrug resistance of S. maltophilia and the increased tolerance to antimicrobial agents of its biofilm cells make the treatment of S. maltophilia infection difficult. The significance of our research is based on understanding the common mechanisms involved in biofilm formation of different S. maltophilia isolates, understanding the diversity of biofilm architectures among strains of this species, and identifying the differently regulated processes in biofilm versus planktonic cells. These results will lay the foundation for the treatment of S. maltophilia biofilms.

## INTRODUCTION

Immunocompromised patients suffer a high risk of nosocomial bacterial infections. These infections are mostly caused by opportunistic bacterial pathogens. Stenotrophomonas maltophilia is a Gram-negative bacterium and belongs to the *Gammaproteobacteria*. The role of S. maltophilia as a causative agent of infection remains unclear, and its pathogenicity is not yet fully understood. Nonetheless, S. maltophilia is today recognized as a clinically relevant human opportunistic pathogen that has been associated with a series of infections, such as respiratory tract infection, surgical site infections, peritonitis, endocarditis, bacteremia, and implant-associated infections (1–3). S. maltophilia together with other major pathogens, such as Staphylococcus aureus, nontuberculous *Mycobacterium*, Pseudomonas aeruginosa, or Burkholderia cenocepacia, can contribute to severe pulmonary infections in cystic fibrosis (CF) patients (4, 5). The prevalence of S. maltophilia in nosocomial infections, particularly lung infections among CF patients, has increased in the last years, and its intrinsic resistance to a broad spectrum of antibiotics makes this organism a relevant multidrug-resistant pathogen in hospitalized patients (1, 6, 7). S. maltophilia exhibits a high level of intraspecies genomic and phenotypic diversity (8–10). Analysis of the globally collected genomes of 1,305 isolates from 22 nations indicated that at least 23 phylogenetically distinct lineages can be observed within the S. maltophilia species (11).

S. maltophilia, like most bacterial pathogens, can adhere and form biofilms on host tissues, such as the respiratory tract and skin, as well as on abiotic surfaces, such as medical devices and implants, and it can cause severe infections (12–14). It is well known that bacterial biofilms provide a shield against antibiotic treatment as well as host defense systems and therefore play a major role in chronic infections (15, 16). Recently, a few molecular keys have been affiliated with S. maltophilia biofilm formation. Among those is the quorum sensing system, which is mediated by the diffusible signal factor (DSF) (17, 18), cell motility (19), and genes involved in lipopolysaccharide/exopolysaccharide biosynthesis (20, 21). In further studies, iron availability was shown to play an essential role in biofilm formation (22, 23).

Within this framework and with respect to the observed genome diversity (11, 24), we asked to what extent genomic diversity affects common phenotypes. Focusing mainly on the biofilm properties of S. maltophilia, we assayed biofilm phenotypes and proteolytic and virulence profiles of selected isolates and correlated these findings with deep RNA transcriptomic data.

Our data provide insights into the biofilm architecture and heterogeneity on the species level. Additional transcriptome data of seven clinical isolates identified major genetic loci differentially expressed in S. maltophilia biofilms. Our findings indicate that a rather small number of genes (9.43% ± 1.36%) are involved in the different lifestyles of biofilm cells compared to planktonic cells.

## RESULTS AND DISCUSSION

### High phenotypic variability at a strain-specific level in S. maltophilia.

**FIG 1:**
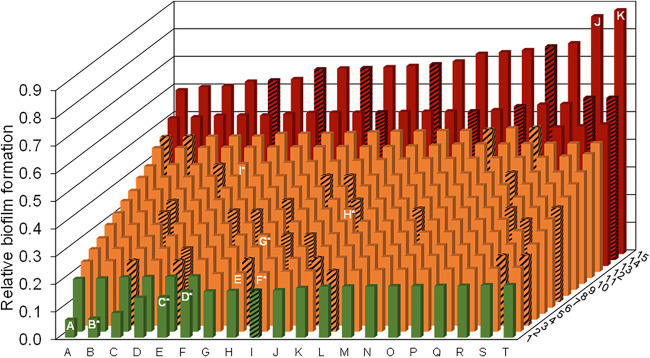
High strain-specific variation with respect to biofilm formation of 300 clinical and environmental isolates of S. maltophilia. Biofilm forming abilities were analyzed in microtiter plates using the crystal violet stain method. Clinical isolates were grown at 37°C (solid bars) and environmental isolates at 28°C (striped bars) for 24 h. Clinical strains PEG 13-85-49 (A), 454* (B), ICU331* (C), SKK55* (D), K279a (E), 677* (F), PC239* (G), PC240* (H), PEG 13–68-68* (I), PEG 13-2-40 (J), and PEG 13-106-64 (K) are highlighted. The last two formed the strongest biofilms. B to D and F to I were included in the transcriptome and other analyses. K279a (E) was included as a control strain. The relative biofilm formation for 6 technical replicates is illustrated. Strains are positioned from low to strong biofilm formers. OD values of relative biofilm formation ranged from 0.064 to 0.88. Isolates with a relative biofilm OD of ≤0.2 were classified as weak biofilm formers (green), and all isolates with a relative biofilm OD of ≥0.5 were classified as strong biofilm formers (red). All isolates with a relative biofilm OD between 0.2 and 0.5 were classified as moderate biofilm formers (orange). Standard deviations ranged from 0.003 to 0.098. Strains employed in transcriptome and virulence analyses are indicated with asterisks. Coordinates of all isolates together with their metadata are listed in Table S1.

**(i) S. maltophilia biofilm formation capabilities vary strongly at a strain-specific level.** Few studies have analyzed the biofilm formation and profiles of S. maltophilia (22, 25). To broaden this approach, we have analyzed the capabilities of 300 clinical and environmental isolates from different European countries to form biofilms (see Table S1 in the supplemental material). Of the tested isolates, 261 were of clinical origin and 39 were of environmental origin. All strains were able to adhere and form biofilms on microtiter plates after 24 h of incubation. Based on the microtiter plate biofilm assay, 14.3% of all the investigated isolates formed strong biofilms, 77.0% formed moderate biofilms, and 8.7% formed weak biofilms (Fig. 1). Thus, biofilm formation varied greatly at a strain-specific level. The relative biofilm optical density (OD) values differed by a factor of almost 14. Interestingly, the commonly used model organism, isolate K279a, was just a moderate biofilm former. With a relative biofilm OD value of 0.211, it was categorized as the second-to-last biofilm former within the category of the moderate biofilm formers.

Notably, we did not observe a relationship between biofilm formation capabilities and the origin of isolation. This concerned environmental as well as clinical isolates. Furthermore, no correlation was observed with respect to the phylogenetic position of the isolates within 1 of the 23 recently discovered lineages of the S. maltophilia species (11).

**FIG 2:**
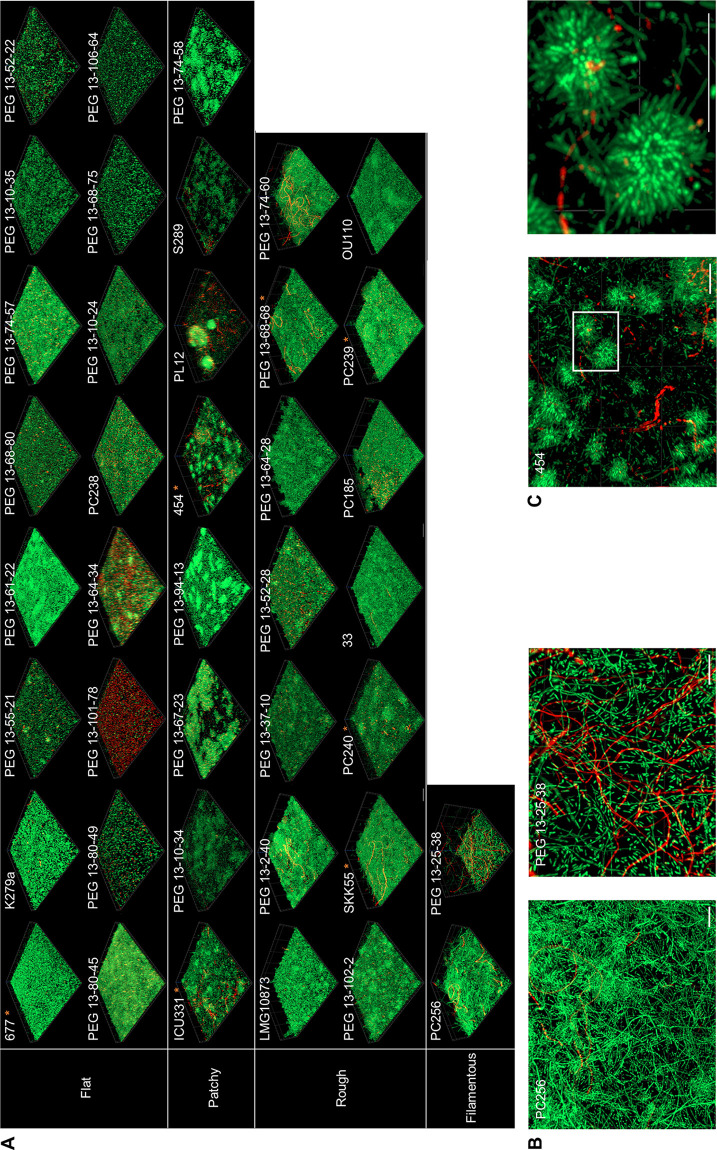
High-level architectural heterogeneity in 40 different clinical S. maltophilia isolates. Biofilm cells were grown under flow or static conditions for 72 h. After a LIVE/DEAD staining, the biofilm architectures were recorded using CLSM. Red, dead cells; green, living cells. (A) The isolates were grouped as forming flat, rough, patchy, and filamentous biofilms based on their overall architectures. Strain identifiers are indicated on the top left corner for each isolate. Strains used in additional transcriptome data are marked with an asterisk. Images represent an area of 100 μm by 100 μm of the respective biofilm. For each of the 40 isolates, at least 3 areas were analyzed. (B) Multicellular and filamentous forms of the isolates PC256 and PEG 13-25-38 are shown via a top view on the biofilm architecture. Scale bar represents 10 μm. (C) Isolate 454 forms rosette-like multicellular clusters of cells. In the right panel, a 4-fold magnification of the boxed area of the left panel is depicted. Scale bar represents 10 μm.

**(ii) High variability in biofilm architecture formed by S. maltophilia.** Acknowledging the various biofilm forming abilities of individual S. maltophilia strains, we went on to systematically evaluate the architecture of the biofilms of clinical S. maltophilia. Therefore, 40 isolates were chosen from the categories weak, moderate, and strong biofilm formers based on the results of our static biofilm assay in microtiter plates (Fig. 1). This analysis further confirmed the high level of strain-specific heterogeneity. Each strain produced a very distinct biofilm architecture, with some showing clear signs of multicellularity (Fig. 2). The architectures observed were categorized into either flat, patchy, rough, or filamentous biofilms. Of the 40 analyzed strains, 16 isolates formed flat (40%), 8 isolates formed patchy (20%), 14 isolates formed rough (35%), and 2 isolates formed filamentous (5%) biofilms. The observed architectures did not differ significantly when strains were grown in flow cells or in static systems. The biofilms ranged in thickness from 2.9 μm (PEG 13-80-49) to 63.6 μm (PEG 13-25-38). Using a LIVE/DEAD stain, the biofilm-grown cells appeared to be viable in most cases. However, a large number of cells of the strains PEG 13-101-78 and PEG 13-64-34 appeared to be dead (Fig. 2A). No relationship between the biofilm architecture of the individual strains and their origin of isolation, their phylogenetic position within the species, or their classification as strong, moderate, or weak biofilm formers was detected. Remarkably, isolate 454 formed dense clusters of cells that appeared to have a rosette-like structure (Fig. 2B and C).

Until now, only a limited number of studies have shown diversity in biofilm architectures among strains of the same bacterial species. A study on the biofilm architectures of 96 Listeria monocytogenes isolates showed variations in biofilm architectures, with a particular predominant morphotype, the honeycomb-like morphotype. However, just like in our observations, there was no correlation between the biofilm architectures and the genetic lineages of the different isolates (26, 27). Furthermore, Hornischer et al. generated a reference database for Pseudomonas aeruginosa and reported on large strain-specific variations with respect to resistance, virulence, and biofilm architecture (https://bactome.helmholtz-hzi.de) (28).

### Proteolytic and virulence profiles differ largely at a strain-specific level.

**FIG 3:**
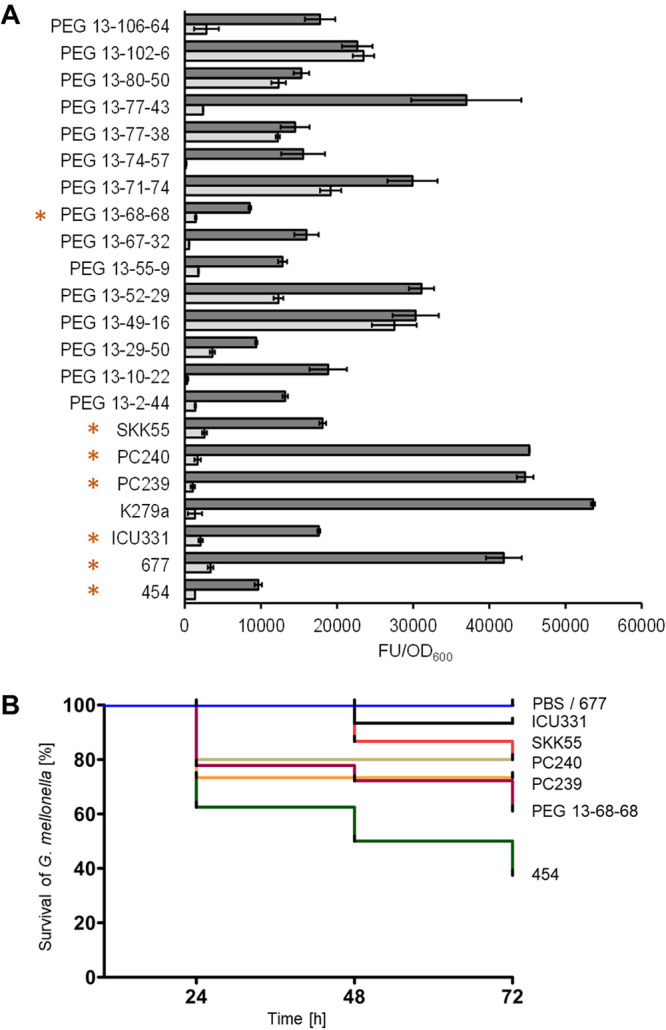
Protease activity and virulence degree vary at a strain-specific level. (A) The extracellular protease activities in biofilm (dark gray) and planktonic (light gray) cultures of 22 clinical isolates were determined in microtiter plates using the EnzCheck protease assay kit. Error bars indicate standard deviations of 3 independent biological replicates. Strains employed in transcriptome and virulence analyses are indicated by an asterisk. (B) The degrees of virulence of S. maltophilia SKK55 (red), 454 (green), ICU331 (black), 677 (gray), PC240 (ochre), PC239 (orange), and PEG 13-68-68 (purple) were tested by recording the survival of Galleria mellonella. PBS (blue) was used as a control. Mortality events were recorded at 24, 48, and 72 h postinfection. FU, fluorescence units.

Knowing that extracellular proteases are associated with virulence of pathogenic bacteria, including S. maltophilia (29–31), and that extracellular proteolytic activity has been associated with biofilm formation in some bacterial species (32), we further asked to what extent the extracellular proteolytic activities of the different isolates differ at a strain-specific level. Therefore, we profiled the extracellular proteolytic activities of several S. maltophilia strains. As assumed, the different strains displayed different levels of protease activity in planktonic as well as in biofilm cultures. Surprisingly, extracellular protease activity was up to 40 times higher in biofilm cultures than in planktonic cultures (Fig. 3A). However, no correlation between biofilm forming capabilities and the phylogenetic origin within the species was observed.

In the light of the above-described observations, we also asked to what extent S. maltophilia isolates differ in their strain-specific virulence patterns. Therefore, we investigated the virulence of seven isolates using the Galleria mellonella model. The 7 isolates were chosen based on their different biofilm architectures, ranging from flat to patchy and rough with filaments. In these tests, we showed that an infection dose of 1 × 10^5^ CFU/larva of the different strains caused a different mortality outcome in G. mellonella larvae. Strain 454 appeared to be most virulent, with only 37.5% of larvae surviving after a 72-h incubation period (Fig. 3B). In contrast, strain 677 showed the least virulence, with 100% survival of larvae after 72 h. The high heterogeneity in virulence of S. maltophilia isolates demonstrated in this study has also been shown in other studies (33, 34). We did not observe a correlation between biofilm profiles, biofilm architectures of these isolates, and their degrees of virulence using the G. mellonella model. However, since we analyzed only seven isolates with respect to their virulence in the G. mellonella model, we cannot exclude that a larger analysis will show that other strains might be more virulent.

### Global transcriptome sequencing (RNA-seq) indicates a set of 106 shared highly expressed genes in S. maltophilia biofilms and not more than 33 strain-specific genes.

To obtain a comprehensive overview of all genes that could possibly play an important role in mature S. maltophilia biofilms, we analyzed the transcriptomes of the same seven clinical S. maltophilia isolates as employed in the Galleria mellonella model (Table 1 and Table S1). The isolates were chosen because they differed in their biofilm architectures (Fig. 2). We first analyzed the transcriptomes of 72-h-old biofilms to identify the most relevant biofilm-expressed genes. We then compared these data sets with transcriptomes of planktonic cultures of the same isolates. Using these comprehensive data sets, we asked three main questions. First, which are the most strongly expressed genes in mature biofilms in general, and what are the main metabolic routes in these biofilms? Second, which are the strain-specifically expressed genes that contribute to the highly heterogeneous biofilm architectures? Third, which are the regulated but commonly expressed genes under biofilm versus planktonic conditions?

**TABLE 1:** Key traits of S. maltophilia clinical isolates included in transcriptome analyses of biofilm and planktonic cells

Strain	Lineage within the species^*a*^	Genome size (Mbp/ORFs^*b*^)	RNA seq (avg total mapped reads)^*c*^		Biofilm architecture/attachment to microtiter plates	No. of genes regulated in biofilm vs planktonic cells^*d*^	Mortality of G. mellonella (%)
			Biofilm	Planktonic			
SKK55	Sm3	4.6/4,296	23,272,784	27,299,628	Rough/weak	284 ↑/34 ↓	20
ICU331	Sm6	5.0/4,716	24,714,069	28,763,824	Patchy/weak	398 ↑/37 ↓	7
677	Sm6	4.7/4,433	22,400,765	8,600,306	Flat/moderate	421 ↑/116 ↓	0
454	Sm6	4.6/4,269	24,759,888	27,668,615	Patchy/weak	338 ↑/67 ↓	63
PC239	Sm4a	4.6/4,349	25,599,936	8,494,898	Flat/moderate	240 ↑/148 ↓	27
PC240	Sm4a	4.8/4,350	28,690,780	9,845,647	Rough/moderate	263 ↑/154 ↓	20
PEG 13-68-68	Sm6	4.5/4,094	24,941,794	9,037,393	Rough/moderate	359 ↑/57 ↓	39

a The genomes of the S. maltophilia isolates were previously sequenced, and pangenome analyses implied that they shared 3,800 orthologous genes/functions with the reference strain K279a (11).

b ORFs, open reading frames.

c The raw reads of the 42 mRNA sequencing runs have been deposited at the SRA nucleotide archive (see Materials and Methods).

d ↑, upregulated; ↓, downregulated.

**(i) S. maltophilia strongly expressed genes in biofilms give insight into metabolism and life on surfaces.** In general, each strain transcribed between 4,000 and 4,350 genes under biofilm conditions (Table S3). It is noteworthy that the genome sizes of the seven isolates varied slightly (Table 1). Using a nucleotide activities per kilobase of exon model per million mapped reads (NPKM) cutoff of 10, in general, 80.5% of all genes were transcribed to some extent under the biofilm conditions (Table S3).

To identify the most relevant and strongly expressed genes in biofilm-grown cells, we decided to focus on the top 250 expressed genes (excluding ribosomal proteins) in our analyses (Table S4). This arbitrarily chosen cutoff correlated in all strains with the expression level of the *rpoD* gene, which is used in many studies as a reference gene (35, 36). This initial analysis included all strongly expressed genes, independent of whether these were regulated or constitutively expressed in biofilm versus planktonic cells. A Venn analysis revealed that 42% (106 genes) of the top 250 biofilm-expressed genes were commonly expressed among all isolates (Tables 2 and 3). Thus, the shared set of 106 commonly expressed genes build a core set of biofilm-expressed genes. These genes are all part of the core genome of the seven isolates.

**TABLE 2:** Shared expressed genes (regulated and nonregulated) among the top 250 expressed genes in the seven analyzed S. maltophilia clinical isolates

Predicted function	454 locus tag (NIPOLPBK no.)^*a*^
Transcription/translation	
Division/cell wall cluster transcriptional repressor MraZ	01488
Flagellar biosynthesis anti-sigma factor FlgM	03920
DNA-binding response regulator	00616
RNA polymerase sigma factor RpoD; RpoH	01893; 01985
HU family DNA-binding protein	02561
DNA-directed RNA polymerase subunit beta′; beta	04131; 04132
RNA polymerase-binding protein DksA	01317
Elongation factor Ts; P	00051; 01310
*O*-Acetyl-ADP-ribose deacetylase	03022
Polyribonucleotide nucleotidyltransferase	03547
DNA recombination/repair protein RecA	04177
DNA starvation/stationary phase protection protein	03142
Adenosylhomocysteinase	01517
Iron aquisition	
TonB-dependent receptor	03236
Energy transducer TonB	02458; 01378
Bacterioferritin	03836
Membrane proteins/transporters	
Porin	03489
MotA/TolQ/ExbB proton channel family protein	02457
DNA transport competence protein ComEA	00199
Biopolymer transporter ExbD	02455; 02456
Cation transporter	03486
Preprotein translocase subunit SecY; SecE	03720; 04138
OmpA family lipoprotein	03078
OmpW family protein	01780
PTS fructose transporter subunit IIA	02219
Peptidoglycan-associated lipoprotein Pal	03125
Tol-Pal system protein YbgF	03126
Membrane protein	03745; 00399; 02118
Glycine zipper 2TM domain-containing protein	01412; 01407; 02726
Ax21 family protein	02718; 00668
Hypothetical proteins/proteins of unknown function	
Tetratricopeptide repeat protein	02459
FAD-binding protein	00093
CBS domain-containing protein	00424
Hypothetical protein	01390; 03621; 03092; 03497; 00615; 00732; 03808; 03451; 02369; 01049; 01025; 02370
Stress response	
Superoxide dismutase	01384
Universal stress protein	00791
Cold shock protein	02817; 03799
Peroxiredoxin	03154
PAS sensor domain-containing protein	03584
Pathogenicity	
Entericidin, EcnA/B family	00629
Respiration/energy	
Cytochrome *bd* oxidase subunit I; II	01342; 01341
ATP synthase subunit alpha; beta; B	00409; 00411; 00407
ATP synthase epsilon chain	00412
Cytochrome *bd*-I oxidase subunit CydX	01340
Adenylate kinase	01697
Azurin	01710
Thioredoxin	01991
Hemerythrin	01154
Protein processing/modification/proteolysis	
Molecular chaperone DnaK; GroEL; GroES	03046; 01940; 01941
FKBP-type peptidyl-prolyl *cis-trans* isomerase	01169
Peptidylprolyl isomerase	03733
Trigger factor	02566
ATP-dependent metallopeptidase FtsH/Yme1/Tma family protein	01006
ATP-dependent Clp protease ATP-binding subunit ClpX	02564
ATP-dependent Clp protease proteolytic subunit	02565
Metabolism/biosynthesis	
Glutamate dehydrogenase	03266
2-Oxoglutarate dehydrogenase E1 component	01425
Pyruvate dehydrogenase (acetyl-transferring), homodimeric type	03384
Lysine decarboxylase	03487
Succinate dehydrogenase flavoprotein subunit	03646
Methylmalonate-semialdehyde dehydrogenase (CoA acylating)	00557
Dihydrolipoyl dehydrogenase	01423
Oxygen-independent coproporphyrinogen III oxidase	01044
Citrate synthase	01750
Dihydrolipoyllysine residue succinyltransferase	01424
Succinyl-CoA ligase subunit beta	02984
Acyl-CoA dehydrogenase	00558
Polyketide cyclase	00315
Alcohol dehydrogenase AdhP	02422
Type I glyceraldehyde-3-phosphate dehydrogenase	01781
Fructose-bisphosphate aldolase class I	03024
Isocitrate dehydrogenase	02640
Malate dehydrogenase	03734
Phenylalanine 4-monooxygenase	02418
Succinate-CoA ligase subunit alpha	02983
Inorganic diphosphatase	01682
Acyl carrier protein	02130
Cell division	
Cell division protein FtsZ	01501

a Locus tags refer to the gene designations GenBank file CP060027 for the isolate 454.

**TABLE 3:** Strain-specific expressed genes (regulated and nonregulated) among the top 250 expressed genes in the seven different S. maltophilia clinical isolates analyzed

Isolate and locus tag^*a*^	Predicted function
S. maltophilia isolate 454	
NIPOLPBK_03560	MerR family transcriptional regulator
NIPOLPBK_03053	Outer membrane protein assembly factor BamE
NIPOLPBK_03052	Ferric iron uptake transcriptional regulator
NIPOLPBK_02989	Pilin
NIPOLPBK_03740	Cell wall hydrolase
NIPOLPBK_02530	C_4_-dicarboxylate transporter
NIPOLPBK_03692	Membrane protein
NIPOLPBK_03349; NIPOLPBK_00082	ABC transporter ATP-binding protein
NIPOLPBK_02384	DNA-binding response regulator
NIPOLPBK_02881	Elongation factor G
NIPOLPBK_00124	TonB-dependent siderophore receptor
NIPOLPBK_03559	Integration host factor subunit alpha
NIPOLPBK_02127	Serine/threonine protein kinase
NIPOLPBK_00518	Thiol:disulfide interchange protein DsbA/DsbL
NIPOLPBK_01192	STAS domain-containing protein
NIPOLPBK_01113	Monothiol glutaredoxin, Grx4 family
NIPOLPBK_00840	SH3 domain-containing-like protein 1
NIPOLPBK_00961	IS1595 family transposase ISAcif2
NIPOLPBK_01249	MexC, transporter periplasmic subunit
NIPOLPBK_03905	Flagellin
NIPOLPBK_02388	Peptide-methionine (*R*)-*S*-oxide reductase
S. maltophilia isolate 677	
FLFIOBJN_01112	Acetyl-CoA carboxylase biotin carrier protein
FLFIOBJN_02765	NADP-dependent malic enzyme
FLFIOBJN_04431	Flagellin
FLFIOBJN_00592	Succinate dehydrogenase iron-sulfur subunit
FLFIOBJN_01823	NADH-quinone oxidoreductase subunit K
FLFIOBJN_01373	GNAT family *N*-acetyltransferase
FLFIOBJN_03492	Single-stranded DNA-binding protein
FLFIOBJN_01536	dTDP-glucose 4,6-dehydratase
FLFIOBJN_01537	Glucose-1-phosphate thymidylyltransferase
FLFIOBJN_01538	dTDP-4-dehydrorhamnose 3,5-epimerase
FLFIOBJN_01823	NADH-quinone oxidoreductase subunit G
FLFIOBJN_01257	Methylisocitrate lyase
FLFIOBJN_01869	Peptidase
FLFIOBJN_02323	Bifunctional proline dehydrogenase/l-glutamate gamma-semialdehyde dehydrogenase PutA
FLFIOBJN_01081	NADP-dependent isocitrate dehydrogenase
S. maltophilia isolate SKK55	
GPNADHDJ_01778	Prepilin-type cleavage protein
GPNADHDJ_02908	Transcription elongation factor GreA
GPNADHDJ_01676	Enolase
GPNADHDJ_01978	PhoH family protein
GPNADHDJ_02891	Long-chain fatty acid-CoA ligase
GPNADHDJ_03134; GPNADHDJ_03863	Acyl-CoA dehydrogenase
GPNADHDJ_03866	TonB-dependent receptor
GPNADHDJ_02768	Cell division protein, ZapA
GPNADHDJ_01975	4-hydroxy-tetrahydrodipicolinate synthase
GPNADHDJ_03864	Alpha/beta hydrolase
GPNADHDJ_04044	Acetyl-CoA *C*-acyltransferase
GPNADHDJ_01835	Sulfoxide reductase catalytic subunit YedY
GPNADHDJ_03735	Cupin domain-containing protein
GPNADHDJ_02081	Fe-S biogenesis protein NfuA
GPNADHDJ_00856	Ax21 family protein
GPNADHDJ_01207	ParA family protein
GPNADHDJ_02348	DNA-binding response regulator
GPNADHDJ_01752	Oar protein
S. maltophilia isolate ICU331	
EIELFIGP_03830	Fis family transcriptional regulator
EIELFIGP_00484	Membrane protein
EIELFIGP_00483	EamA family transporter
EIELFIGP_03092	ACR protein
EIELFIGP_04252	IMP dehydrogenase
EIELFIGP_01515	Translational throttle protein EttA
EIELFIGP_00265	RNA helicase
EIELFIGP_02438	AcrB/AcrD/AcrF family protein
EIELFIGP_03678	TonB-dependent receptor
EIELFIGP_03829	Type II toxin-antitoxin RelE/ParE family toxin
EIELFIGP_01306	*S*-Adenosylmethionine decarboxylase proenzyme
EIELFIGP_01865	F_o_F_1_ ATP synthase subunit A
EIELFIGP_04217	Transcriptional regulator
EIELFIGP_03352	Aspartate-semialdehyde dehydrogenase
EIELFIGP_03290	Flagellin
EIELFIGP_03291	Flagellin
S. maltophilia isolate PC239	
PLCFDHLH_00300	Transcriptional regulator
PLCFDHLH_01540	Classical arabinogalactan protein 4
PLCFDHLH_01650	Helix-turn-helix domain-containing protein
PLCFDHLH_04366	Penicillin-binding protein activator
PLCFDHLH_03625	Dihydrolipoyllysine-residue acetyltransferase
PLCFDHLH_03306	Iron-sulfur cluster assembly accessory protein
PLCFDHLH_03284	methyltransferase domain-containing protein
PLCFDHLH_03397	Fimbrial biogenesis outer membrane usher protein
PLCFDHLH_04557	IS3 family transposase
S. maltophilia isolate PC240	
IMCGPPIG_03647	RidA family protein
IMCGPPIG_02449	GlsB/YeaQ/YmgE family stress response membrane protein
IMCGPPIG_00866	TonB-dependent receptor
S. maltophilia isolate PEG 13-68-68	
AEPCKKLL_01703	Polyketide cyclase
AEPCKKLL_03165	Pilin
AEPCKKLL_02474	Molybdopterin molybdenum transferase MoeA
AEPCKKLL_03864	Flagellar hook protein FliD
AEPCKKLL_02476	Molybdopterin converting factor subunit 2 protein
AEPCKKLL_00278	Ubiquinone/menaquine biosynthesis *C*-methyltransferase UbiE
AEPCKKLL_01787	TonB-dependent receptor

a Locus tags refer to the GenBank files: SKK55, CP060025; ICU331, CP060026; 677, CP060024; 454, CP060027; PC239, CP060023; PC240, CP060022; and PEG13-68-68, CP060021. Hypothetical proteins are not included.

**FIG 4:**
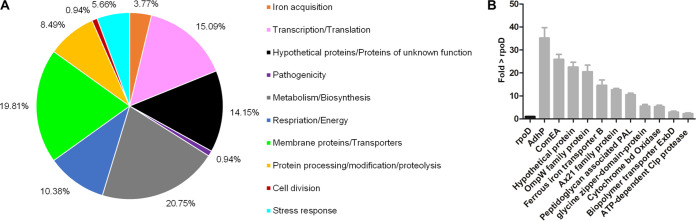
Global transcriptome analysis of seven biofilm-grown S. maltophilia isolates, SKK55, 454, ICU331, 677, PC240, PC239, and PEG 13-68-68. (A) Functional distribution of the 106 commonly expressed genes (regulated and not regulated) among the top 250 strongly expressed genes in the biofilms of the seven clinical isolates (Table 2). Expression data were extracted from global RNA-seq analyses, and the top 250 strongly expressed genes are listed in Table S4. (B) Mean fold change in relation to the NPKM value of the housekeeping gene *rpoD*. Error bars indicates standard deviations and are based on three independent biological replicates.

A more detailed functional analyses indicated that among the 106 shared genes, the largest fractions were assigned to metabolism (20.75%), to membrane proteins and transporters (19.81%), and to transcription and translation (15.09%) (Fig. 4A and Table 2).

Surprisingly, all strains expressed a gene coding for the alcohol dehydrogenase AdhP at a high level, up to 38 times higher than the expression level of the housekeeping gene *rpoD* (Fig. 4B). In Escherichia coli, AdhP is probably involved in the production of propanol, or similar short-chain alcohols, under biofilm conditions (37). Since this enzyme belongs to the alcohol dehydrogenase superfamily, we speculate that it is further involved in the production of various short-chain alcohols in S. maltophilia. In this respect, it had not been reported that S. maltophilia produces primary or secondary alcohols. Therefore, we analyzed the supernatants of biofilm and planktonic cultures using ^1^H nuclear magnetic resonance (NMR). The NMR data indicated that biofilm-grown cells produced micromolar amounts of 1- and 2-propanol as well as acetate together with several unknown compounds (Table 4 and Fig. S1). These short-chain alcohols were not observed in any of the planktonic cultures. The occurrence of the different alcohols, however, varied at a strain-specific level.

**TABLE 4:** Chemical compounds identified in biofilm and planktonic culture supernatants of seven different S. maltophilia clinical isolates

Isolate	Growth condition	Compound detected and verified^*a*^				
		1-Propanol	2-Propanol	Threonine	Acetate	Ethanol
SKK55	Biofilm	X	X	X	_	X
	Planktonic	_	_	_	_	X
ICU331	Biofilm	_	_	X	X	X
	Planktonic	_	_	_	X	_
454	Biofilm	_	X	X	X	X
	Planktonic	_	_	_	X	?
677	Biofilm	_	_	X	X	X
	Planktonic	_	_	X	X	X
PC239	Biofilm	_	_	X	X	X
	Planktonic	_	_	X	_	_
PC240	Biofilm	X	X	X	X	X
	Planktonic	_	_	_	_	_
PEG 13-68-68	Biofilm	_	_	X	X	X
	Planktonic	_	_	_	_	X

a Representative spectra of the different compounds in the supernatants are given in Fig. S1. X, detected/verified; -, not detected/verified.

Furthermore, it is noteworthy that the genes coding for a Pal protein together with the YgbF protein were strongly transcribed in all seven characterized strains. The Pal protein is part of the Tol-Pal complex and is involved in cell envelope-related processes. It is involved in transport processes but also in release of outer membrane vesicles and cell septum formation (38) and has been associated with survival and pathogenesis in bacteria (39).

Among the other most relevant genes, many are involved in iron uptake, such as the gene coding for the biopolymer transporter ExbD. Moreover, all strains expressed genes of the ATP-dependent Clp protease complex, which is commonly involved in proteolysis and regulation of different metabolic processes. Interestingly, some studies have shown that the Clp protease complex also plays a role in biofilm formation and virulence in Listeria monocytogenes and Actinobacillus pleuropneumoniae (40, 41).

The gene encoding the DNA transport competence protein ComEA was strongly expressed in all seven isolates. ComEA is essential for DNA transport and bacterial competence (42). We have recently identified the *comEA* locus as potentially involved in phenotypic heterogeneity in S. maltophilia (10). The observation that *comEA* is highly expressed may suggest a broader role in S. maltophilia biofilm formation (43).

**(ii) Genes strongly expressed at a strain-specific level give the first clues on heterogeneous phenotypes.** Altogether, 142 of the 250 most strongly expressed genes seemed to be strain-specifically expressed in only one of the seven analyzed isolates. While strain PC240 expressed only five genes at a strain-specific level, 454, the most virulent strain (Fig. 3B), expressed 33 genes at a strain-specific level (Table 3). While we initially hoped that these data would give us clues about the different biofilm architectures, the data did not allow for a final conclusion on these highly diverse biofilm phenotypes. Nevertheless, some of the observed differential expression profiles may in part provide the first clues about the high strain-specific variability.

For instance, SKK55 biofilms and some others consisted of a remarkable number of multicellular structures, such as long filamentous cells (Fig. 2). The *zapA* gene was one of 30 strain-specific genes of SKK55 (Table 3), which is involved in cell division (44–46). The elevated expression of the *mraZ* gene has been linked to the occurrence of filamentous cells in E. coli and *Mollicutes* (47, 48). The observation made in this study may explain the frequent occurrence of filamentous cells in S. maltophilia SKK55 biofilms.

**(iii) Relatively few genes may play a role in matured biofilms of S. maltophilia.** In addition to comparing the transcriptomes of the seven S. maltophilia isolates under biofilm conditions, we also compared biofilm and planktonic cells for each isolate (Fig. S2). Generally, under planktonic conditions, 96.9% of all genes were transcribed (NPKM value > 0) (Table S5) and 3.0% were not transcribed in all seven strains. Remarkably, the majority of the top 250 strongly expressed genes in biofilms were equally highly expressed in planktonic cultures. These were often essential genes involved in general and aerobic metabolism (Tables S4 and S6).

**FIG 5:**
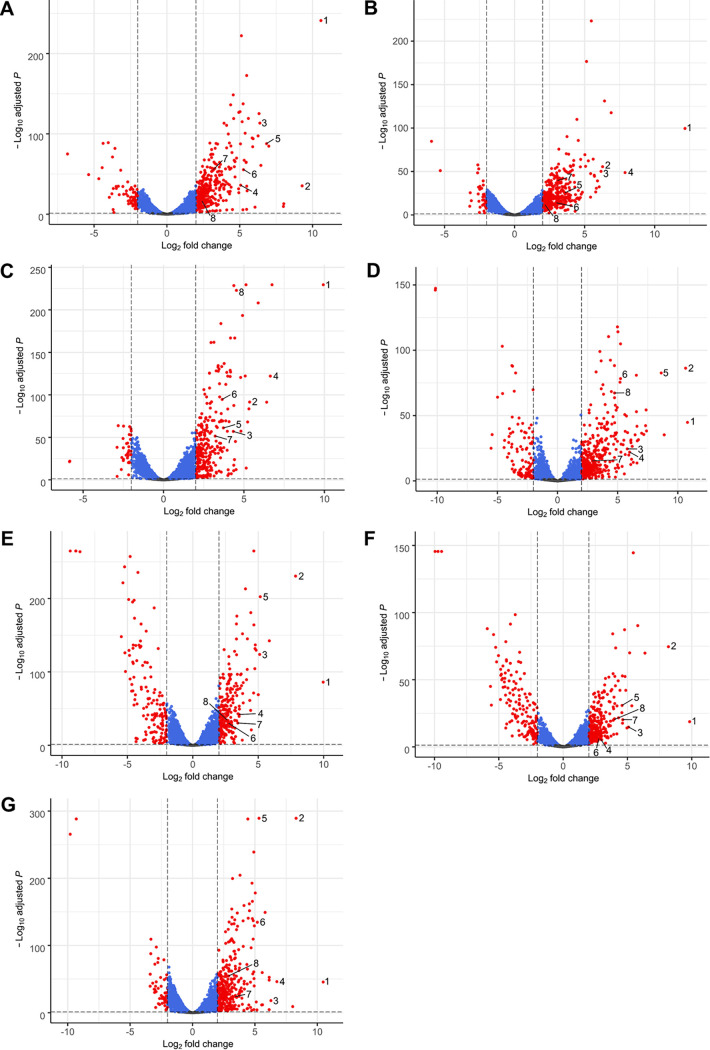
Differentially expressed genes between biofilm and planktonic cells of clinical S. maltophilia isolates. Shown are volcano plots of differentially expressed genes between biofilm and planktonic cells of S. maltophilia 454 (A), ICU331 (B), SKK55 (C), 677 (D), PC239 (E), PC240 (F), and PEG 13-68-68 (G). The 8 strongest commonly upregulated genes in all isolates are indicated by numbers as follows: 1, hypothetical protein (NIPOLPBK_02286); 2, PAS sensor domain-containing protein (NIPOLPBK_03584); 3, TonB-dependent receptor (NIPOLPBK_02287); 4, hemin uptake protein HemP (NIPOLPBK_01535); 5, sulfite reductase flavoprotein alpha subunit (NIPOLPBK_03585); 6, TetR/AcrR family transcriptional regulator (NIPOLPBK_02786); 7, cytochrome *b* (NIPOLPBK_03926); and 8, flagellin (NIPOLPBK_03906). Locus tags were derived from the 454 genome entry CP060027 and homologues retrieved from the corresponding genomes of the other isolates (see Table 1 for GenBank accession numbers). Genes without significant regulation (*P*_adj_ > 0.05 [gray]), genes with *P*_adj_ of <0.05 (blue), and significantly up- or downregulated genes (*P*_adj_ < 0.05; log_2_ fold change of greater than 2 or less than −2 [red]) are illustrated. For isolate 454, 338 genes were upregulated of a total gene count of 4,269 genes, while 67 genes were downregulated. Of a total of 4,716 genes, 398 genes were upregulated and 37 genes were downregulated in ICU331. For SKK55, 284 genes were upregulated and 34 genes were downregulated of a total of 4,296 genes. In 677, 421 of 4,433 genes were upregulated, while 116 were downregulated. For PC239, 240 genes were upregulated and 148 genes were downregulated of a total of 4,564 genes. Of a total of 4,564 genes, 262 genes were upregulated and 153 genes were downregulated in PC240. For PEG 13-68-68, 359 genes were upregulated and 57 genes were downregulated of a total of 4,094 genes. Normalized gene read counts were used to build volcano plots.

Comparing the biofilm-expressed genes with the planktonic expressed genes in detail, we noted that just 9.43% ± 1.36% of all genes were differentially regulated, consisting of 7.46% ± 1.49% upregulated genes and 1.96% ± 1.02% downregulated genes using log_2_ fold change cutoffs of 2 and −2 and a *P* adjusted (*P*_adj_) value of ≤0.05 (Fig. 5 and Table 1). In general, between 240 and 421 genes were upregulated and between 34 and 154 genes were downregulated when we compared the planktonic with the biofilm cells (Table 1).

**FIG 6:**
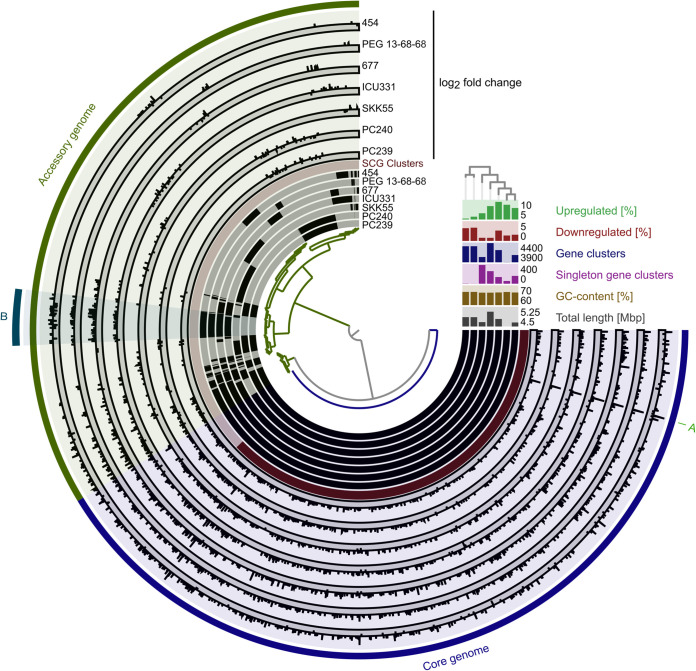
Pangenome and differential gene expression analysis of seven clinical S. maltophilia isolates. Comparative Anvi’o pangenome analysis of seven clinical S. maltophilia isolates combined with differential gene expression of biofilm versus planktonic cells of these isolates was conducted. The dendrogram in the center represents the relationship between 5,881 gene clusters (GCs) involving 30,168 gene calls. The seven inner layers represent individual genomes, which are compared to each other. In the layers, black indicates the presence of gene clusters and gray their absence. The genomes are organized regarding the presence/absence of GCs as indicated by the phylogenetic tree in the top right. The red layer represents the single gene copy (SCG) clusters, in which dark red indicates the presence and light red the absence of SCG clusters. The next seven layers represents the log_2_ fold change of gene clusters differently regulated in biofilm versus planktonic cells of individual isolates. The dark gray sublayer indicates the downregulated gene clusters and the light gray ones the upregulated gene clusters in biofilm cells, respectively. The core (blue) and accessory genomes (green) are indicated in the next layer. In the outermost layer, some interesting regions are highlighted (A and B). (A) Hypothetical protein (NIPOLPBK_02286 in strain 454). (B) Among others, several nitrate assimilation-related genes. The right-hand side section reveals the genome length, the GC content, the number of gene clusters present in just one genome, the total gene cluster number, and the proportions of up- and downregulated genes for each isolate.

**FIG 7:**
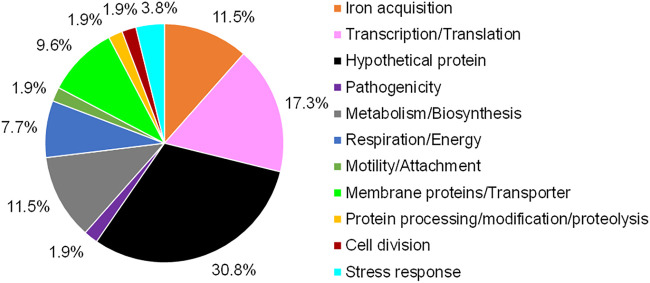
Functional distribution of commonly upregulated genes in biofilm cells of seven clinical S. maltophilia isolates. Isolates used for the transcriptome analysis were the clinical isolates SKK55, ICU331, 454, 677 PEG 13-68-68, PC239, and PC240. Fifty-two commonly upregulated genes (Table 5) were identified by a Venn analysis and classified by the function of their gene products.

A pangenome analysis of all seven isolates identified the gene clusters present in the core genome (Fig. 6). The integration of the log_2_ fold change values of all up- and downregulated genes into the pangenome analysis revealed that 68% ± 8% of all regulated genes belong to the core genome. A Venn analysis of all gene clusters of the regulated genes generated by the pangenome analysis identified 52 genes that were commonly upregulated and 1 gene (5S rRNA) that was commonly downregulated in all isolates (Table 5). The largest fraction of the commonly upregulated genes coded for hypothetical and uncharacterized proteins (30.8%), followed by genes involved in transcription and translation (17.3%) (Fig. 7). A remarkably high number of genes involved in iron acquisition (11.5%) were found to be upregulated in almost all seven analyzed S. maltophilia isolates (Fig. 7 and Table S7).

**TABLE 5:** Commonly up- and downregulated genes in biofilm versus planktonic cultures of seven S. maltophilia clinical isolates

Predicted function	454 locus tag (NIPOLPBK no.)^*a*^	Log_2_ fold change
Commonly upregulated genes		
Transcription/translation		
TetR/AcrR family transcriptional regulator	02786	5.26
Ribosomal subunit interface protein	02220	3.62
Glycine cleavage system regulatory protein	03619	4.56
30S ribosomal protein S4	03723	4.17
DNA-binding transcriptional regulator Fis	01972	4.94
YncE family protein	02887	3.70
Transcriptional regulator	01212	4.22
Zinc finger domain-containing protein	02534	3.93
Ribonucleotide diphosphate reductase subunit beta	02059	5.45
Iron acquisition		
TonB-dependent receptor	02380; 02287	2.30; 6.38
Hemin uptake protein HemP	01535	5.06
Iron-regulated lipoprotein	03483	5.89
Energy transducer TonB	01848	5.44
TonB-dependent receptor	02972	2.69
Membrane proteins/transporters		
Glycine zipper 2TM domain-containing protein	02726	5.60
PTS fructose transporter subunit IIA; IIBC	02219; 01596	4.10; 2.62
Lysine transporter LysE	00300	5.48
Carbohydrate porin	01597	4.10
Hypothetical proteins/proteins of unknown function		
Hypothetical protein	01621; 02971; 01805; 03497; 00009; 00662; 03451; 02369; 00474; 00227; 01538; 03808; 02285; 01411; 03807; 02286	2.36; 2.48; 2.18; 2.86; 8.03; 2.50; 3.40; 3.98; 3.75; 4.24; 2.80; 5.12; 5.92; 5.85; 4.68; 10.57
Pathogenicity		
Putative protein YqiC	03780	4.62
Respiration/energy		
Cytochrome *b*	03926	3.20
FMN reductase	01620	2.34
Ferredoxin-NADP reductase	01395	2.34
NADH-quinone oxidoreductase subunit A	03525	4.68
Stress response		
PAS sensor domain-containing protein	03584	9.29
Peroxiredoxin	03321	5.48
Protein processing/modification/proteolysis		
Copper chaperone	01272	5.17
Metabolism/biosynthesis		
Sulfite reductase flavoprotein subunit alpha	03585	6.84
Isopenicillin N synthase family oxygenase	03202	2.20
Ribonucleoside-diphosphate reductase subunit alpha	02058	3.97
*N*-Acetyltransferase	00115	3.32
Methylthioribulose 1-phosphate dehydratase	01261	3.48
Acyl-CoA dehydrogenase	03947	3.15
Cell division		
Classical arabinogalactan protein 4	03411	6.26
Motility/attachment		
Flagellin	03906	2.41
Commonly downregulated genes		
5S rRNA	03698	−6.82

a The above-listed locus tags refer to the GenBank file CP060027 for isolate 454.

Most interestingly, a hypothetical gene which is the corresponding homologue of *smlt2713* in strain K279a was the most strongly and differentially expressed gene in all strains, with a log_2_ fold change of 10 to 12 (Fig. 6, region A, and Table S7). It carries a TbpB (transferrin-binding-protein) motif, implying that it is possibly involved in iron uptake. Notably, Tbp proteins have been associated with virulence in Actinobacillus pleuropneumoniae (49, 50). It is known that iron plays a role in biofilms of Bacillus subtilis (51) and S. maltophilia (22) as well as boosts biofilm formation of Staphylococcus aureus (52), P. aeruginosa (53), and Campylobacter jejuni (54). Further, a sulfite reductase gene was also upregulated in biofilm cells of all seven isolates, implying that the ability to assimilate sulfate and sulfite for energy conservation appears to be present in this organism. Furthermore, genes involved in nitrate transport and reduction were upregulated in four of the seven tested isolates (454, 677, ICU331, and PEG 13-68-68 [Fig. 6, region B]). This suggests an anaerobic mode of respiration in biofilm cells. Anaerobic respiration in biofilms has also been reported for P. aeruginosa (55, 56). Surprisingly, many motility affiliated genes were transcribed at elevated levels (Table S7). Domka and colleagues also demonstrated that the expression of some motility-related genes was increased in 7- to 24-h-old biofilms of Escherichia coli (57). Further studies have reported the involvement of flagella and pili in shaping the biofilm structures of P. aeruginosa and E. coli (58–61). Thus, our findings may imply a similar function in S. maltophilia.

Furthermore, all isolates carry three copies of the *ax21* genes, whereas for the reference strain K279a the presence of just two copies is known (*smtl0387* and *smtl0187*). While two copies appeared to be strongly transcribed in biofilm cells of almost all seven isolates, a comparison with the planktonic cells implied that they are differentially transcribed in a strain-specific manner. In four of the seven tested isolates, the transcription of one or both copies of the *ax21* gene was upregulated in biofilm cells (Table S7). The expression of the *ax21* copies seems to be dependent on quorum sensing via the DSF in S. maltophilia (62). Nonetheless, its direct role in the biofilm formation by S. maltophilia remains unclear.

Notably, all analyzed S. maltophilia isolates code for at least four different cytochrome *c* oxidases. Under hypoxic conditions in biofilms, some of these cytochrome *c* oxidases were upregulated in some of the 7 isolates (Table S7), and a common cytochrome *b* oxidase was commonly upregulated in all isolates (Table 5).

Overall, with a proportion of 1.96% ± 1.02%, relatively few genes were downregulated in all seven investigated isolates. Generally, tRNAs and 5S and 16S rRNAs were downregulated in almost all analyzed isolates (Table S7). This indicates a reduced translation in biofilm cells in comparison to planktonic cells, which was already demonstrated, for instance, for Staphylococcus epidermidis, Clostridium perfringens, and Gardnerella vaginalis (63–65).

Furthermore, some genes of a not-yet-characterized operon stretching from NIPOLPBK_01784 to NIPOLPBK_01792 (using the 454 nomenclature) were downregulated in all isolates. This operon codes for three hypothetical proteins, one membrane protein, one putative protease, two TldD proteins, and one MoxR-like-ATPase. TldD family proteins inhibit the DNA gyrase (66), and the MoxR-like-ATPase has chaperone-like functions (67). Moreover, a lysoplasmalogenase gene (NIPOLPBK_00344 in 454) was downregulated in four of the seven isolates. These enzymes form fatty aldehyde and glycerophosphoethanolamine or glycerophosphocholine by cleaving the vinyl ether bond of lysoplasmalogen (68, 69). These findings may imply a novel role of fatty acid and glycerol derivatives in the planktonic lifestyle.

Finally, on average, 1.16% ± 0.78% of all genes were strain-specifically upregulated and 0.56% ± 0.37% downregulated in the seven studied isolates. The majority of these genes are affiliated with metabolism (Table S8).

### Conclusions.

Our study analyzed in detail a large number of S. maltophilia isolates with respect to biofilm forming capabilities, architecture, and metabolite production in biofilms, together with protease profiling and estimation of virulence in the G. mellonella model. Together, our data imply that within isolates of the species S. maltophilia an unexpected high phenotypic diversity exists. While strain specificity is a major challenge in S. maltophilia clinical research, we nevertheless have identified a set of 106 commonly and strongly expressed genes in biofilms and a maximum of 33 strain-specific genes.

Further, our data imply that, on average, 7.46% ± 1.49% of all genes were upregulated in biofilms versus planktonic cells and, on average, 1.96% ± 1.02% of all genes were downregulated.

The phenotypic and omics data generated in this study will provide a solid basis for further S. maltophilia biofilm studies and will help to reveal potential targets for the development of more effective drugs against this emerging pathogen.

## MATERIALS AND METHODS

### Bacterial strains, chemicals, and growth conditions.

Table S1 summarizes the S. maltophilia clinical and environmental isolates used in this study together with their metadata.

S. maltophilia strains were routinely cultured in LB medium (10 g/liter of tryptone, 5 g/liter of yeast extract, and 5 g/liter of NaCl) at 28°C or 37°C if not otherwise stated.

### S. maltophilia biofilm assays.

**(i) Static biofilm assay in microtiter plates.** For analyses of static biofilms, S. maltophilia cells were grown in flat-bottomed microtiter plates (Nunc MicroWell, catalog no. 161093; Thermo Fisher Scientific, Waltham, MA) according to the method of Steinmann et al., with the following modifications (24). An overnight culture of S. maltophilia was adjusted to 4.0 × 10^7^ cells/ml in LB medium. A total of 200 μl of the culture was pipetted in microtiter plates and incubated without shaking at 37°C for 24 h. After drying the biofilm, the adhered cells were stained with 50 μl of 0.5% crystal violet solution per well for 5 min and were washed three times with water afterwards. The plates were dried for 30 min at 37°C before crystal violet was dissolved in 150 μl of 33% acetic acid per well and the optical density at 595 nm (OD_595_) of the dye was measured. Six technical replicates were done per strain or condition. To obtain the relative biofilm OD value, we first measured the total OD (growth) value of each well in our microtiter plate assay and then set the crystal violet value in ratio to this, that is, value OD_595_ crystal violet/OD_600_ growth = relative biofilm OD value. Isolates with a relative biofilm OD of ≤0.2 were classified as weak biofilm formers, and isolates with a relative biofilm OD of ≥0.5 were classified as strong biofilm formers. All isolates between an OD of 0.2 and 0.5 were classified as moderate biofilm formers.

**(ii) Cultivation of S. maltophilia biofilms in flow chambers or μ-slides.** For analyses of the biofilm architecture, S. maltophilia isolates were cultivated in three-channel flow chambers (70) or eight-well μ-slide (ibiTreat, catalog no. 80826; ibidi USA Inc., Fitchburg, WI). All experiments were performed at 28°C with 10% LB medium. After 72 h, cells were stained using the LIVE/DEAD BacLight bacterial viability kit (Thermo Fisher Scientific, Waltham, MA) and the biofilm was analyzed by confocal microscopy.

### Fluorescence imaging analysis of S. maltophilia biofilms.

Visualization of flow chamber and μ-slide biofilms was performed using a confocal laser scanning microscope (CLSM) Axio Observer.Z1/7 LSM 800 with airyscan (Carl Zeiss Microscopy GmbH, Jena, Germany) and a C-Apochromat 63×/1.20W Korr UV VisIR objective. The microscope settings for the different fluorescent dyes are shown in Table S2. The analysis of the CLSM images and three-dimensional reconstructions were done with ZEN software (version 2.3; Carl Zeiss Microscopy GmbH). Biofilm architecture was analyzed at least at three different positions for each strain, and one representative CLSM image was chosen. More detailed quantitative analyses of biofilm architecture, such as roughness and thickness, were done using BiofilmQ software version 0.1.4. (71) (https://drescherlab.org/data/biofilmQ/docs/usage/installation.html).

### Galleria mellonella virulence assay.

The virulence of seven S. maltophilia isolates was tested using the Galleria mellonella model (72, 73). Final-instar larvae of G. mellonella were obtained from Biosystems Technology, Exeter, UK. Healthy larvae were identified by their cream color without dark discoloration and their vital movements. Prior to infection, the S. maltophilia isolates were grown in LB medium at 37°C to an OD_600_ of 0.5. Cells were washed twice in phosphate-buffered saline (PBS) and adjusted to 1 × 10^7^/ml. For each isolate, an inoculum of 1 × 10^5^ CFU/larva was then injected into the last proleg of the larvae. For each experiment, a total of 15 larvae were used for each isolate. A total of 15 larvae injected with 10 μl of PBS each served as a control. The larvae were then placed on Whatman paper-lined petri dishes and incubated at 37°C. The larvae were observed at 24, 48, and 72 h. Larvae were considered dead when no movement and a complete dark discoloration were observed.

### Assessment of the extracellular proteolytic activity of biofilm and planktonic cultures.

For assessing the protease activity, we used the method as previously published by Steinmann et al., with minor modifications (24). For planktonic cultures, 30 ml of 10% LB (DWK Life Sciences GmbH, Westfalen, Germany) was inoculated with overnight cultures and placed to shake at 28°C to an OD_600_ of 0.5. Ten-milliliter aliquots of the cultures were pelleted at 4°C for 20 min. The supernatant was collected and sterile filtered (CA membrane, 0.2 μm). For biofilm cultures, overnight cultures were adjusted to an OD_600_ of 0.05 in 10% LB medium. The biofilm of four biological replicates was grown in 24-well microtiter plates (Nunc cell culture plate, catalog no. 142475; Thermo Fisher Scientific, Waltham, MA) at 28°C for 48 h. The supernatant was collected, centrifuged as described above, and sterile filtered. The protease activity was determined using the Molecular Probes EnzChek protease assay kit (Thermo Fisher Scientific). The protease activity of three technical replicates was measured in black BRANDplates microtiter plates with transparent bottoms (BRAND GmbH + Co. KG, Wertheim, Germany). A total of 100 μl of substrate, 90 μl of digestion buffer, and 10 μl of culture supernatant were pipetted into each well. Plates were incubated at 37°C and fluorescence was measured every 30 min in a Synergy HT plate reader (BioTek Instruments Inc., Winooski, VT) at 590/20-nm extinction and 645/40-nm emission wavelengths.

### RNA-seq and data analysis.

RNA-seq was done as previously published, with minor modifications (74). For the preparation of cell material for RNA-seq analyses, biofilm cells were grown in flow cells as described above at 28°C for 72 h using 10% LB medium. To avoid contamination, media were supplemented with 50 μg/ml of ampicillin. Biofilms were grown in flow cells for 72 h and were washed out of the flow cells with a 20% stop mix (95% ethanol and 5% phenol) and pelleted at 4°C for 20 min. Planktonic cells were grown in 10% LB medium supplemented with 50 μg/ml of ampicillin at 28°C and were pelleted at the exponential phase (OD_600_ ∼ 0.5) at 4°C for 20 min. The pellets were frozen in liquid nitrogen for later analysis. Three biological replicates of each strain were prepared.

Harvested biofilm/planktonic cells were resuspended in 800 μl of RLT buffer from the RNeasy minikit (Qiagen, Hilden, Germany) with β-mercaptoethanol (10 μl/ml), and cell lysis was performed using a laboratory ball mill. Subsequently, 400 μl of RLT buffer (RNeasy minikit) with β-mercaptoethanol (10 μl/ml) and 1,200 μl of 96% [vol/vol] ethanol were added. For RNA isolation, the RNeasy minikit was used as recommended by the manufacturer, but instead of RW1 buffer, RWT buffer (Qiagen, Hilden, Germany) was used to also isolate RNAs smaller than 200 nucleotides (nt). To determine the RNA integrity number (RIN), the isolated RNA was run on an Agilent Bioanalyzer 2100 using an Agilent RNA 6000 Nano kit as recommended by the manufacturer (Agilent Technologies, Waldbronn, Germany). Remaining genomic DNA was removed by treating the samples with Turbo DNase (Thermo Fisher Scientific, Waltham, MA). Pan-Prokaryozes riboPOOL kit v1 (siTOOLS Biotech, Planegg/Martinsried, Germany) was used to reduce the amount of rRNA-derived sequences. For sequencing, the strand-specific cDNA libraries were constructed with a NEBNext Ultra II directional RNA library preparation kit for Illumina (New England BioLabs, Frankfurt am Main, Germany) using 50 ng of rRNA-depleted RNA and 8 PCR cycles. To assess the quality and size of the libraries, samples were run on an Agilent Bioanalyzer 2100 using an Agilent high-sensitivity DNA kit as recommended by the manufacturer (Agilent Technologies, Waldbronn, Germany). The concentrations of the libraries were determined using the Qubit double-stranded DNA (dsDNA) HS assay kit as recommended by the manufacturer (Life Technologies GmbH, Darmstadt, Germany). Sequencing was performed by using the HiSeq4000 or HiSeq2500 instrument (Illumina Inc., San Diego, CA) using either the HiSeq 3000/4000 SR cluster kit or the HiSeq Rapid PE cluster kit v2 for cluster generation and the HiSeq 3000/4000 SBS kit (50 cycles) for sequencing in the single-end mode and running 1 × 50 cycles. For quality filtering and removal of remaining adaptor sequences, Trimmomatic v-0.39 (75) and a cutoff phred-33 score of 15 were used. The mapping of the remaining sequences was performed with the Bowtie program (version 2) (76) using the implemented end-to-end mode, which requires that the entire reads align from one end to the other. Surviving reads were mapped against the reference genomes. NCBI GenBank numbers are listed at the end of the Materials and Methods section and in the footnote to Table 1. Reads per gene feature were counted using feature Counts v.2.0.0 (77). Subsequent analysis was performed in R (version 3.6.1) (78), including normalization of the reads with DeSeq2 (version 1.26.0) (79) using the default fold change-shrinkage algorithm. The rlog function with “blind” parameter set to TRUE within DeSeq2 was used for data transformation to generate principal-component analyses (PCAs), which were drawn with ggplot2 (version 3.2.1) (80), whereas the Enhanced Volcano packages (version 1.4.0) (81) were used for the creation of volcano plots.

### Venn analysis.

Proteins with an identity of > 90% were combined in one cluster. Venn Painter tool (version 1.2.0) (82) was used to construct the Venn diagram and to identify the commonly expressed or regulated genes between the analyzed isolates.

### Pangenome analysis.

Pangenome analysis and the illustration of the corresponding differential gene expression were conducted with Anvi’o 6.1 (83). Gene clusters were built with a minbit of 0.5. For the differential gene expression, gene clusters with a log_2_ fold change of greater than 2 were illustrated as upregulated and gene clusters with a log_2_ fold change of less than −2 were illustrated as downregulated.

### Determination of metabolites via ^1^H NMR analyses.

For NMR analysis, the supernatant of biofilm cultures grown in flow cells for 72 h in 10% LB medium at 28°C was collected. Samples were mixed with 40 mM phosphate buffer (K_2_HPO_4_ and KH_2_PO_4_ in H_2_O) in a ratio of 1:9 (sample to buffer). A standard (trimethylsilylpropanoic acid [TMSP], 5 mM) was also added. The ^1^H NMR samples were measured with an excitation sculpting sequence for water suppression with a 600-MHz spectrometer with 128 scans at 300 K. A total of 65,536 data points and a spectral width of 16.0221 ppm was acquired with the O1 at the signal of water at 2,819 Hz. All spectra were processed with Topspin version 4.0.8, applying an exponential multiplication of the free induction decay (FID) with a line broadening factor of 0.3 Hz.

### Data availability.

The raw reads of the 42 mRNA sequencing runs have been deposited in the SRA nucleotide archive under BioProject no. PRJNA646397 and correspond to the SRA accession numbers SRX8752168 to SRX8752209. Updated GenBank files of the genomes of the seven isolates included in the transcriptome analyses were submitted under the following identifiers (IDs) and accession numbers: UHH_SKK55, CP060025; UHH_ICU331, CP060026; UHH_677, CP060024; UHH_454, CP060027; UHH_PC239, CP060023; UHH_PC240, CP060022; and UHH_PEG13-68-68, CP060021.

## Supplementary Material

Supplemental file 1

Supplemental file 2

Supplemental file 3

Supplemental file 4

Supplemental file 5

Supplemental file 6

Supplemental file 7

Supplemental file 8

Supplemental file 9

Revised Table S1
