# Supplementary material for: Phenotypic and Transcriptomic Analyses of Seven Clinical Stenotrophomonas maltophilia Isolates Identify a Small Set of Shared and Commonly Regulated Genes Involved in the Biofilm Lifestyle
Source: Appl Environ Microbiol. 2020 Nov 24;86(24):e02038-20. doi: 10.1128/AEM.02038-20 (PMC7688217; doi:10.1128/AEM.02038-20)
Supplement: Supplemental file 1 [file AEM.02038-20-s0001.pdf]

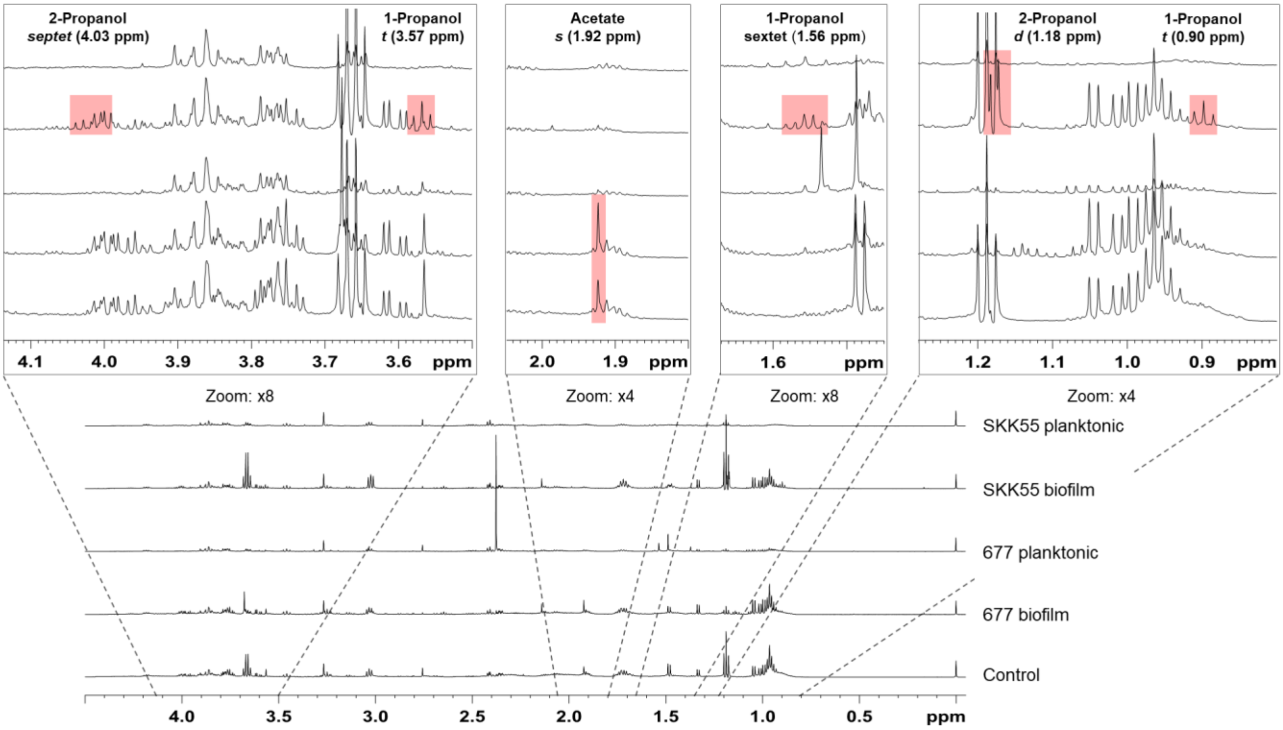

FIGURE S1: [1] H-NMR spectra (600 MHz) of the supernatant of planktonic and biofilm cultures of isolates SKK55 and 677. Prior to measurement, the samples were mixed with 40 mM of phosphate buffer (1:9). The spectra were recorded at 300 K and measured with water suppression (excitation sculpting). The zoom factor of the cutouts was set to four and eight times. 677 showed an increase of acetate (s, 1.92 ppm) compared to the control, which was 10 % LB. SKK55 showed new signals of 1-propanol (*t*, 3.57 ppm, 6.8 Hz; *sextet*, 1.56 ppm; *t*, 0.90 ppm, 7.6 Hz) and 2-propanol (*septet*, 4.03 ppm, 6.3 Hz; *d*, 1.18 ppm, 6.3 Hz).

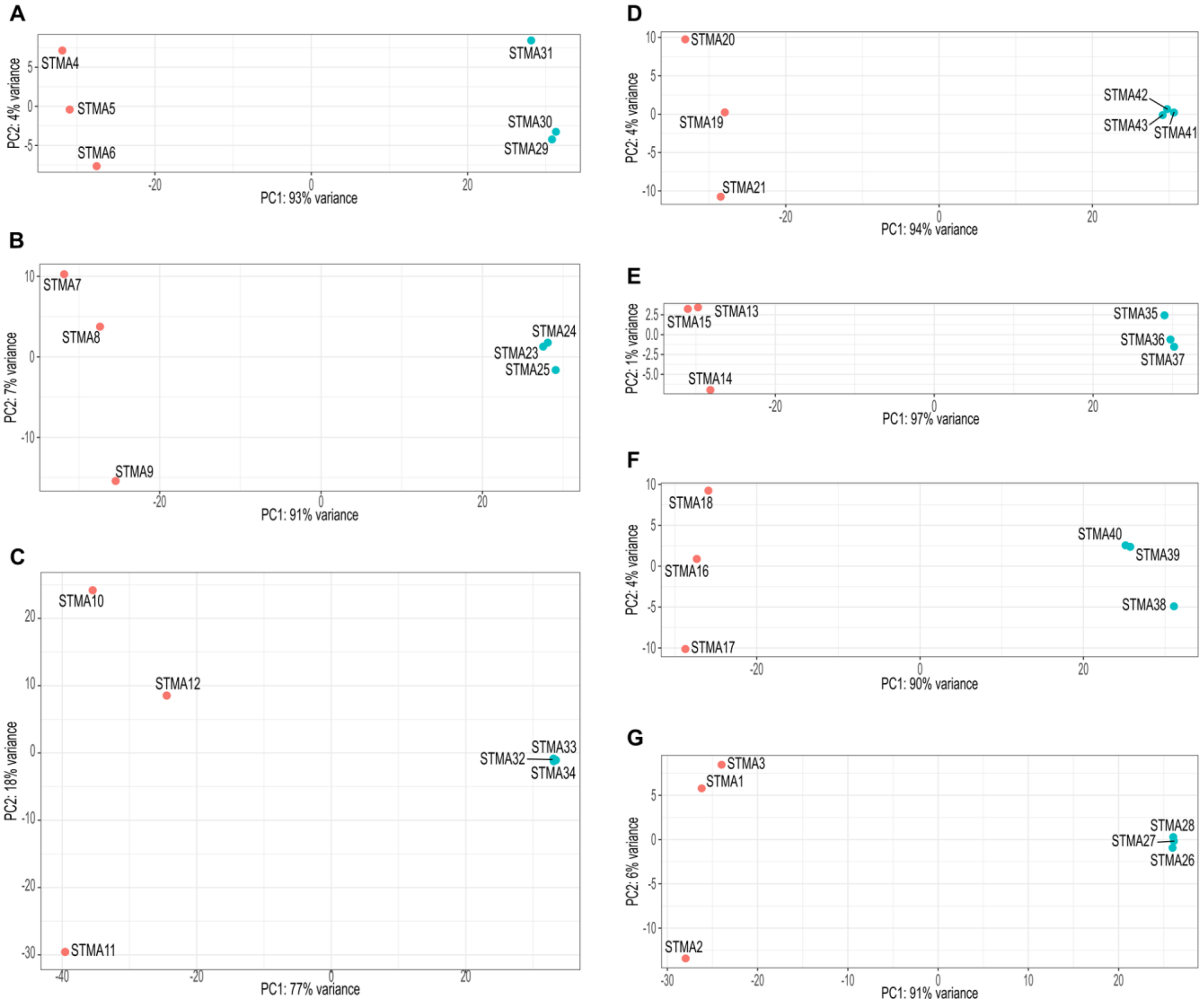

FIGURE S2: Principle component analysis (PCA) plots of transcriptome analysis of seven clinical *S. maltophilia* isolates. PCA plots represent each three biological replicates of biofilm (red) and planktonic (blue) samples of *S. maltophilia* 454 (**A**), ICU331 (**B**), 677 (**C**), PEG 13-68-68 (**D**), PC239 (**E**) and PC240 (**F**), SKK55 (**G**) for transcriptome analysis. Plots reveals a high similarity between the replicates of the respective samples, but a significant difference between samples of planktonic and biofilm cells. Plots were generated with the rlog function in DeSeq2.
